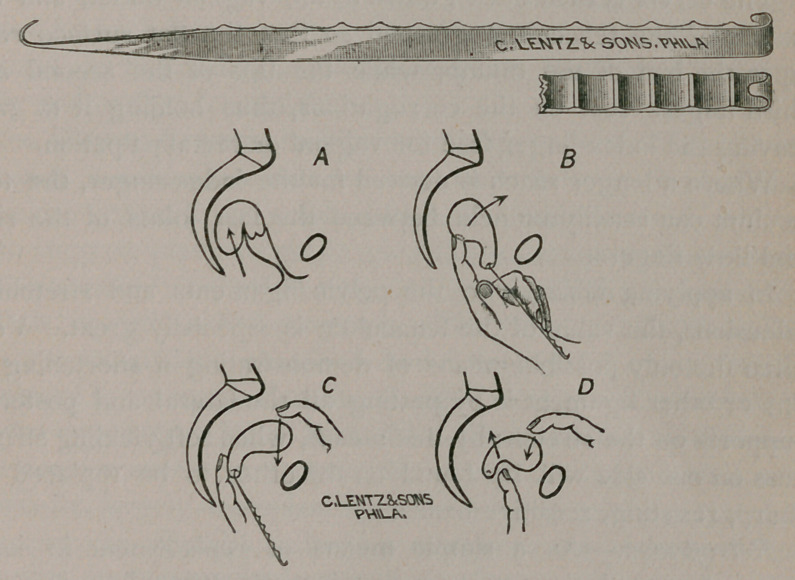# Philadelphia Obstetrical Society

**Published:** 1889-07

**Authors:** J. M. Baldy

**Affiliations:** Secretary; 328 South Seventeenth Street


### Society Reports.

### PHILADELPHIA OBSTETRICAL SOCIETY.

FRIDAY, May 3, 1889.

THE President, Dr. THEOPHILUS PARVIN, in the chair.

Dr. B. C. Hirst: Laparotomy for Intestinal Obstruction.

The following case is reported, not because it is an especially rare and interesting one, but because it so clearly emphasizes the importance of early operative interference whenever indubitable symptoms of intestinal obstruction manifest themselves.

Mrs. F., æt. 46; seen in consultation with Drs. Prendergast and Ziegler; she has always been constipated; about five days before I saw her, she had taken a number of aperient pills, as she had not had a passage for some days before. The medicine was without effect in moving the bowels, but caused intense abdominal pain. Three days afterward stercoraceous vomiting began. The gentleman in charge of the case had injected large quantities of water into the bowels without result; no purgative medicine, however, had been administered. We finally agreed to try an injection of glycerine. This was followed by the evacuation of quite a large portion of well-formed fæces. Hoping that we had at last overcome the obstruction, concentrated solution of salts was administered in small doses frequently repeated. This had had barely time to act when profuse fæcal vomiting again appeared. An operation was then, of course, determined upon and performed late at night, with the valuable aid of Dr. Jos. Price's technical skill and good advice, assisted also by Drs. Prendergast and Ziegler. The small intestine was occluded by a mesenteric band so tightly that not a particle of fæces could go through. The proximal portion was enormously distended and congested; the distal portion for the length of about eight inches was almost black in color, and looked already gangrenous. The band was easily divided. The distended gut was punctured, and a basin full of liquid fæces evacuated. The opening was most carefully sewed up. The discolored portion of the gut was watched for about twenty minutes, while the circulation gradually returned to it, and its appearance much improved. The abdominal wound was then closed and dressed. The whole operation, including the time consumed in watching the strangulated portion of intestine, lasted but thirty minutes. The woman's condition was fairly good afterward, but she soon began to sink, and died of exhaustion thirty-six hours later. The case was complicated by the worst nurse, in the person of a religious sister and relative of the patient, that I ever came in contact with.

Had the operation been undertaken as soon as stercoraceous vomiting appeared, in all probability the woman's life would have been saved. The delay, however, under the circumstances, was natural, and the belief that the obstruction had been overcome seemed justified. The lesson, I think, that this case should teach,—the lesson, at least, that it has taught me,—is to open the abdomen as soon as stercoraceous vomiting occurs; the operation may occasionally prove unnecessary, but then little harm will have been done; more often it will rescue the individual from impending death.

DR. H. A. KELLY.—I recently operated upon a similar case, *in extremis*. An old woman had had stercoraceous vomiting for many days, and was prostrated with a tympanitic abdomen, a very rapid pulse, and a deeply furred, brown tongue.

I found, at the abdominal section, that the small gut was contracted down into a small cord from the ileo-cæcal valve eighteen inches up.

A prolonged, waiting, do-nothing policy in the hands of an ignorant practitioner had done the work.

If the operator is to give himself and the patient a chance, these cases must be seen early.

DR. JOHN B. DEAVER.—I have seen a number of cases of obstruction of the bowel, and it is astonishing with what freedom physicians will administer aperients in these cases. Dr. Hirst speaks of giving concentrated solution of salts. I recently saw a case where the physician had given six drops of croton oil, and wondered why he did not get any movement of the bowels.

Treves, who has written the best article on this affection, plainly tells us that there are cases which call for large doses of opium from the commencement.

In regard to stercoraceous vomiting, I do not think that it is safe to wait for this symptom. We know that the most common form of intestinal obstruction, in the adult at least, is strangulation by a band. The next most frequent form is volvulus, and the most common seat of volvulus is at the sigmoid flexure. In these cases it is rather the exception to have stercoraceous vomiting until the patient is *in articulo mortis*. In the majority of cases where you have obstinate vomiting not allayed by simple measures, and with this constipation and the evidences of depression, these symptoms will warrant the surgeon in operative interference.

DR. J. PRICE.—I am surprised to hear a surgeon say that there is ever an indication for opium in bowel obstruction. I have seen a large number of these cases, but I have never recognized an indication for opium save in a dying patient. It is just these large doses of opium which render the surgeon absolutely powerless. Scarcely a surgeon dares work after a large dose of opium has been given. He is called to see a patient where the physician suspects obstruction. He finds a comfortable patient with all the symptoms masked. It would be impossible to induce such a patient to submit to operation, the only thing that will save his life. Diagnosis should always be made, where possible, before administering an opiate.

The case reported by Dr. Hirst has many points of interest. It was unquestionably a chronic case of obstruction. The bowels were filled with fæces and gas. It is in these cases where the patients are dying from fæcal poisoning and shock, that we must minimize every detail in our operation. They cannot stand a prolonged operation and manipulation of vital viscera like the intestines. Nor will the condition of the viscera permit the introduction of sutures. The suture tracts will become gangrenous. The operation must be performed as quickly as possible. Sometimes you can do nothing more than make an artificial anus with drainage of bowel contents. In this way you may save life and be able to perfect the cure at a later period. The methods of Senn and one or two gentlemen in New York, by means of disk and ring methods for forming intestinal anastomoses, are valuable and rapid, and will no doubt contribute much to perfect intestinal surgery.

DR. J. C. DA COSTA.—With all due deference to want of success Dr. Price has had with opium, I may say that Mr. Treves is not entitled to the credit of this treatment. If the old editions of Gross' surgery are examined, it will be found that Prof. S. D. Gross recommends opium in intestinal obstruction, and states that a number of cases have recovered under it.

DR. J. M. BALDY.—With all due respect to Dr. Gross and to Mr. Treves, I do not think that we need be proud of the treatment of intestinal obstruction by opium. We know that intestinal obstruction has a greater mortality than any other abdominal disease. The mortality is simply frightful. Treves mentions two thousand cases as occurring in England alone. The use of opium in intestinal obstruction tends to increase the distension and obstruction already existing; and if the degree of obstruction was such that it might have been relieved, it spoils the chance. In addition, no one can make a correct diagnosis with a patient stupified with opium.

I agree with Dr. Deaver that we ought not to give purgatives. If injection will not relieve the case quickly, we should not wait beyond obstinate vomiting, and stercoraceous vomiting should be absolutely the last symptom that we should wait for.

DR. JOHN B. DEAVER.—With all due respect to Dr. Baldy and Dr. Price, I cannot agree with them. This is an important and practical point, especially important to the young practitioner. Opium is called for until the diagnosis is made. In all of these cases of intestinal obstruction, it is difficult to make the diagnosis. We know that the paroxysmal pain of intestinal obstruction is due to peristalsis. We know that opium is not the cause of the distension; this is due to the obstruction. Where we have active obstruction, any form except *ileus paralyticus*, opium is of service. Whereas I am in favor of the administration of salines in ordinary abdominal surgery, I cannot think that they are called for until we have made up our mind whether or not we have intestinal obstruction.

DR. J. PRICE.—I have a case which I think will demonstrate the mistake of Dr. Deaver. Day before yesterday my brother was asked to see a patient in whom he suspected intestinal obstruction. He forbade absolutely the administration of opium for the relief of the paroxysms of pain, and gave large enemata. He obtained nothing more than a colic movement. In less than six hours fæcal vomiting occurred, and three or four hours later he did a section for the relief of a strangulated bowel. The bowel was almost gangrenous at points. Salines were administered and four movements followed. The temperature is now normal. The pulse under 90. This was an ideal case, and demonstrates the importance of a clear diagnosis and early work before the patient is dying of fæcal poisoning, collapse, and peritonitis. It matters not who first used opium, the cases all died just the same.

DR. BARTON C. HIRST.—I agree with the criticism in regard to the administration of purgatives in these cases. We did not give the concentrated solution of salts until we thought that the obstruction had been relieved. It was given with the view of getting rid of the accumulated fæces. If there had been any idea that obstruction still existed, it would not have been employed.

DR WILSON reported an intra-ligamentous cyst.

This specimen was removed from a woman named Mary W., æt. 32, unmarried, and a native of Scotland. She commenced to menstruate at 13 years of age, and was always regular. She had never submitted to coitus. Six months before examination, she first noticed a slight tumor the size of a goose's egg in her right side. A careful bimanual examination under ether revealed a large cyst on the right side of the uterus, and apparently situated in the broad ligament. The diagnosis of intra-ligamentous cyst was made by my colleague, Dr. Noble, and myself, he using the alternate diagnosis of fibrocystic tumor of the uterus. This cyst had grown rapidly, as in my experience all intra-ligamentous cysts do. Five days after admission, and ten days after last menstrual flow, the abdomen was opened by a small incision one and one-half inches in length. The diagnosis verified the broad ligament incised on its anterior surface, and the cyst easily removed by first aspirating its contents and then sponging out the empty sac from its incasement in the broad ligament. The case offered no difficulties, and was principally remarkable for the ease with which the cyst was enucleated. But two or three arterial twigs required pressure with the hæmostatic forceps, and but one was tied with a ligature. This case is reported in order to show with what facility these cysts may be diagnosed and operated upon. Also to show the advantage gained in the freedom from hemorrhage where the sac is separated from its attachments by the adhesions being gently sponged away. This method has the additional advantage that hemorrhage can be readily and instantly detected by its employment, and the dangers of the operation thereby materially decreased. The woman made an excellent and uninterrupted recovery, the sutures being removed on the sixth day, and the woman discharged from the hospital on the twelfth day. No drainage was employed in this case. In both cases I am thankful to have had the assistance of my colleague, Dr. C. P. Noble, and my friend and resident doctor, Francis Weidned.

DR. BARTON C. HIRST.—I had a case presenting nearly the the same features as that reported by Dr. Wilson, and in which I had adopted the same procedure. I opened the abdomen and evacuated the abscess externally at the point found most convenient. I could get no history, and do not know whether the abscess followed confinement or not, but I think not. The case made a complete recovery. I agree with the recommendation of abdominal section to determine the proper point of opening in these large abscesses about the lumbar muscles. If I had attempted to open this abscess without previous abdominal section, I should very likely have opened the peritoneal cavity. There is usually, too, some doubt as to the diagnosis of these cases. In the one just referred to it was uncertain whether the tumor was an osteo-sarcoma of the pelvic bones, or a deep-seated abscess; the section cleared up all doubt.

DR H. A. KELLY exhibited Eastman's Clamp Forceps for securing the broad ligaments in vaginal hysterectomy, and made some remarks upon the simplified technique of the operation in the use of clamps instead of ligatures.

Also a new CORRUGATED TENACULUM (*v.* Fig.) designed to afford a third hand to the operator. This tenaculum, which I exhibit for the first time this evening, has opened up new avenues for me in the successful diagnosis and treatment of abnormal pelvic conditions.

*Diagnostically*, it is of value in bringing the uterus down towards the pelvic outlet, displacing all structures attached to the uterus in a downward direction, clearing the hollow of the sacrum for bimanual palpation.

This is effected by holding the cervix down near the vaginal outlet by means of this tenaculum, *with the same hand which is being used in vaginal and rectal palpation*, while the free abdominal hand assists in palpating and bringing the structures to be questioned within reach.

The tenaculum made for this purpose is eighteen centimeters in length and seven millimeters in breadth in the handle.

The end of the handle is recurved into a small, short, hook, giving a purchase to the hand for traction.

The other end tapers off into the stout hook, which is caught in the cervix when the instrument is in use.

The handle is flattened on its upper surface, which is divided into thirteen shallow gutters, one centimeter in width.

When in use, the hook is guided by the index-finger into the cervical canal, which it enters, where it is firmly fixed by pressing it firmly upwards into the anterior lip of the cervix.

The cervix is then drawn down to the vaginal outlet, and the handle of the tenaculum grasped so that the flat surface rests upon the ball of the thumb, while the tips of the second and third fingers rest on the corrugations, thus holding it *in situ*, leaving the index-finger free for vaginal or rectal palpation.

Where a longer reach is desired for the index-finger, the tenaculum can readily be held between the last joints of the ring and little fingers.

In applying *massage* to the pelvic ligaments and stretching adhesions, the value of the tenaculum is especially great. Very often the only possible means of demonstrating a shortening of one or other ligament is by putting all the lateral and posterior supports on the stretch; by this means, when soft,yielding structures on one side will be found on the other to be replaced by sharp, resisting, sensitive bands.

*Retroflexion.*—As a simple means of replacement in some cases of retroflexion and retroversion, the tenaculum is especially useful.

Here, sometimes, it is absolutely necessary, in order to replace the uterus satisfactorily, to exert pressure and traction in three directions at one time.

The great obstacle in the way of an easy and immediate reposition, in cases of retroflexion where the cervix has not also descended towards the vaginal outlet, is the fact that upward pressure upon the fundus through the vagina or rectum only tends to drive the body of the uterus into the body of the sacrum, as indicated by the direction of the arrow in Fig. A.

By catching the tenaculum in the cervix and drawing it down, the uterine body is straightened out; then holding the tenaculum as shown in Fig. B, and exerting pressure upon the fundus with the index-finger of the same hand introduced into the rectum, the body yields and begins to go upward in the pelvis, describing an arc around the fixed cervix, sweeping clear of the sacral promontory ; at this point the fundus is caught by the other free hand working through the abdominal walls, and drawn forward (*v.* Fig. C). The lower hand now lets go the tenaculum, and the index-finger is used to push the cervix high up and far back into the pelvis, while the body is brought further forward and forced down. In this way the reposition is satisfactorily and easily accomplished.

DR. HENRY BEATES, Jr.—In submitting a report of the following case, a somewhat detailed account will be given, because there are two or three points which, if closely studied, will, at least, suggest questions challenging the accuracy of notions prevailing among "abdominal surgeons," upon which are based diagnosis, procedure, and prognosis. The merely mechanical skill necessary to achieve what is termed a "successful operation" is possessed by so many, that on all sides we hear of removal of uterine appendages for all sorts of functional and organic diseases. The acquiescence of patients to the attendant's suggestion is, undoubtedly, largely dependent upon the assurances of the operator that recovery offers by so large a margin as six, eight, and ten per cent., and immunity from suffering by somewhere near ninety-five per cent. 'Tis well enough to cite an operator's report that one hundred and fifty or more consecutive successful operations have been performed; but what is implied by such statements ? In this city alone, analysis of the town recovery is demonstrated to mean, *escaped death from operation to live on suffering from abdominal hernia and fistula, fæcal fistula, neuralgia and disability.* Our literature teems with such narratives, and what does it mean ? Simply that our knowledge is utterly inadequate to warrant this precipitate rashness, both of operative procedure and prognosis, and that there is demanded a knowledge which will promise, at least, the ability to know when *not* to operate. Subordinate questions present at the table, involving most profound responsibility, such as the removal of a healthy tube and ovary in cases of unilateral pyosalpinx, because there is a prevalent belief that pus in the tube means gonorrhœal infection, and where this radical measure has not been done, subsequently the untouched structures were affected and a second operation required. That such occurrences eventuate no one for a moment questions; but whether pyosalpinx *is* invariably due to gonorrhœa and cannot follow other processes is by *no means settled.* Again, take a pus-tube; in some we have caseous metamorphosis of the whole structure, while in another there is simply retained pus, the tube being distended but not organically altered. Does this suggest any thought? Before we unhesitatingly advise radical measures, let us first know whether our notion is an *ignis fatuus*, or a well-matured and scientifically proven fact. The course of salpingitis, when considered as a disease *per se*, is not clearly and definitely known, and there seems to be no effort to classify the symptomatology, and, in a word, its natural history, into recognized pathological conditions. If the question, does pyosalpinx with caseous metamorphosis of the whole tube belong exclusively to the gonorrhœal type be propounded, is there a valid reply? Is it known whether pyosalpinx without this change is an inflammation of another type, or, considering both propositions, are there not cases in which the healthy side remains unaffected if left, and if so, why? Then the alternatives form a group of propositions; *i. e.*, whether in certain conditions it is not better to allow non-interference, because of possible after-results, such as fistula and remaining unbenefited. The case I narrate might be contributory to a definite classification, but for incidental conditions which, unfortunately, invalidate any such deductions; and it is for this fact, as well as throwing light upon one cause of abdominal fistula, that I presume upon the society's time.

In November, 1886, I was asked by an abdominal surgeon to see a patient in an eleemosynary institution. Right pyosalpinx was diagnosticated, and her poorly nourished, anæmic and illy-developed condition attributed to the long suffering incident to the affection. She had a high temperature range, and was in agony. The condition, we had every reason to infer, was due to gonorrhœa. The operator removed the affected tube, which was filled with pus and caseous, as well as the ovary, which had undergone cystic changes. The apparently healthy left tube and ovary were allowed to remain. Patient, after three weeks of diminished suffering, was permitted to sit up, and in another was returned from a private institution where she had been placed, to increase chances of recovery, to the original habitat. Here she was immediately, in her enfeebled condition, set the task of scrubbing, with cold water, tin utensils. The condition of the endometrium I do not know; neither do I know whether it had received any special treatment; but certain it is that salpingitis promptly developed coincidently with the chilling. How this was treated I do not know; but in a few weeks I was asked to see the case, and found the left tube and ovary matted together with inflammatory deposit, and the patient suffering intensely. A second operation was decided upon, and when the abdomen was opened, it was decided that the pyosalpinx could not be removed. Patient recovered from this, and was discharged. Having no home, friends secured a residence, where for weeks she suffered from increasing fever and repeated attacks of pelvic peritonitis. She applied to me, begging that an operation be tried, and in April of 1888, a third laparotomy was done, and the specimen here shown removed. Indications demanded drainage. The tube (glass) was allowed to remain five days. The site of tube failed to heal, and an abdominal fistula was present. This received all sorts of treatment with no relief. Circumstances resulted in her entering the Pennsylvania Hospital, where as many opinions and plans of treatment were adopted as there were physicians. Some blamed the long time which the tube was allowed to remain, others suggested that silver wire was left in abdomen, a few believed carelessness in antisepsis to have been the cause, and so on *ad infinitum*. Finally the patient was discharged, and existed with this painful complication until December of same year, when worn out with suffering and prevented from supporting herself, she submitted to a fourth operation. The fistula was, under ether, explored and found to be a Y-shaped channel, the stem of which traversed the space between the peritoneum and transversalis fascia, and led to the seat of original disease. This greatly surprised me, as I certainly expected to find it due to the extensive manipulation required in the third operation. The arms of the fistulous tract extended along the cicatrix, upward toward umbilicus and downward toward pubis. This whole tract was thoroughly explored and scraped. At the base of the stem was a small piece of sponge, probably there from treatment of the external orifice, having worked its way down. In addition to this was a suppurating granular surface which was all removed with a curette. The result proved that the fistula was due to a suppurating surface, as after removal of this it promptly healed. This fistula then was *not* due to too long use of drainage-tube; and this important factor in treatment should not, from the extremely narrow ground of a single case, be undervalued and hastily condemned. Since this case, I carefully explore fistulous tracts, have had two, and find the condition due to a suppurating surface, which has either been left at time of operation or subsequently developed. Let not the drainage-tube receive the censure which frequently belongs to a hurried or perhaps incomplete operation. Where no such conditions obtain, I have yet to see a fistula from a tube. Now as to the pathology. That gonorrhœa played an important role cannot be questioned; but when the proposition presents whether the healthy tube and ovary, under similar conditions, should be removed, certainly such a case as this one cannot supply positive evidence, for too many influences of unknown power exist and invalidate any conclusion, whether *pro* or *con*. It is this perplexing condition which determines me to report the case, and have an opportunity of listening to the opinions, as above facts moved them, which this society may offer.

DR. H. A. KELLY.—I think that this is quite a remarkable case. I do not know of a parallel one. Both the operator and the patient are to be congratulated on their courage. The treatment of fistula is an important matter, and it is one which every abdominal surgeon will have to consider at one time or another. It is impossible to have a large series of cases without meeting with some which require drainage, and where, if it be an abscess, the septum between the abscess and the rectum is very thin. It is impossible to prevent the formation of a fistula by the subsequent breaking down of that septum. I have seen several such cases. They have all run a more or less protracted course, but in the end have gotten well. In one case I had a ureter cyst, due, I think, to puncture of the ureter by the needle used in passing a deep suture in the floor of the pelvis. I have known of cases of infection of ligatures from a tube left in for a protracted length of time. I do not say too long, because it is often necessary to leave the tube in for a long time. I have frequently had ligatures sent to me by patients. In all these cases the fistulæ which have persisted for weeks or months have healed when the ligature has been rejected.

DR. LONGAKER.—I wish simply to emphasize the point that the drainage-tube is not always the cause of fistula. In one case in which the tube was kept in only thirty-six hours, there is still a fistula; the operation was done four months ago.

DR. J. PRICE.—I cannot permit the statement of a member of the Obstetrical Society, that he has repeatedly had ligatures returned to him by patients, to go out without a remark. Such has not been my experience, and I think that it has not been Dr. Goodell's. I should like to hear from him on this point. I have never had a ligature come away. The ligatures used are often too large. Such a ligature is dangerous, because its size prevents good tying. I have before called attention to the fact that it is common for patients to die of hemorrhage. Almost weekly we have in this city patients dying on the table, or shortly after the operation. Deaths from hemorrhage are exceedingly common, and in many cases the hemorrhage is due to a large ligature. Where the patient does not die, the ligature almost always comes away. In one case, where the patient had suffered for a long time, Dr. Penrose reopened the abdomen, and found an enormous ligature tied with enormous knots. The patient had submitted to a number of operations for the relief of a fæcal fistula. If sufficiently small and well tied, the ligatures should never come away. I have had a large experience with unhealthy conditions in the pelvis, and have never had a ligature come away, nor have I had fæcal fistulæ or fistulous openings; nor have I ever had a ligature sent me by a patient after recovery.

DR. J. M. BALDY.—I think that Dr. Kelly did not say that all fistula cases were due to the drainage-tube. I believe that many cases are due to the tube. I do not believe that it is always the result of previous infection of the ligature. I have used the same ligature from the same reel on two cases within fifteen or twenty minutes of each other, and in one case fistula occurred, while the other patient made an uninterrupted recovery. The one with fistula had a drainage-tube used, and the other did not. In regard to large ligatures, I do not believe that such a ligature as Dr. Price exhibited at the County Medical Society is necessarily the cause. In a number of cases I have used this large plaited ligature. In none of these cases was there any accident whatever. I have had three fistulæ. Two followed supra-pubic hysterectomy, and the third was a case in which I assisted Dr. Weeks. It was a case of abscess requiring prolonged drainage for three or four weeks. I referred to this case in my paper a few weeks ago. Since that time a ligature has come away. The ligature is of the smallest size that can be used. The ligature was tied with the Tait knot, and shows the three loops very distinctly.

From the fact that I have used large ligatures with no accident, and have met with fistula after the use of a small ligature, and again using the same ligature on two cases side by side, and having fistula in one and not in the other, leads me to think that it is not the fault of the ligature. I think that we must look for other causes; I believe the tube is one of the most prolific of these causes.

DR. J. PRICE.—The ligature used by Dr. Baldy is the large twisted ligature. I am sure that he has never used the large plaited ligature to which I have referred. So much silk is a foreign body, and I have never known it not to come away.

DR. WILLIAM GOODELL.—The subject of fistulæ following laparotomy is a very perplexing one to me. So far as I recollect, I have never had fistula after oophorectomy. All of the fistulæ have occurred in the bad cases of ovariotomy and intraligamentary cysts. As Dr. Kelly remarks, I do not see how it is possible to avoid the occurrence of fistula where you have a tumor closely fastened to the rectum. I have had a patient return me two ligatures. This was in one of my earlier operations, an oophorectomy done *per vaginam*. An abscess formed and the two ligatures were thrown off. I have on several occasions fished ligatures out of a fistulous track, and thus cured it. I am satisfied that one patient on whom I performed ovariotomy a year ago and who is suffering from a fistula, has a ligature at the bottom of the track.

I am so sure that the drainage-tube plays an important role in many of these cases, that I always deem it a misfortune when I have to leave one in for any length of time.

Over a month ago I operated in a neighboring city on an ovarian tumor. It was a case of malignant cyst, and all of the abdominal organs were infected with papillomata. I removed the tumor, which was a large one and colloid in its character. On the right side I found a papillary mass involving the tube, ovary and womb, which I made no effort to remove. A glass drainage-tube was put in, and kept in for a week. I then suggested to the physician in charge that a rubber tube be substituted. Yesterday I received a note from him stating that there was evidently an urinary fistula. But I think that the tube in this case had nothing to do with the formation of this fistula, and that the opening into the bladder is the result of the extension of the diseased cancerous mass in the right groin.

I have not had a large number of fistulæ, but all of them have given more or less trouble. I have a fistula in a case in which I removed an intra-ligamentary cyst sometime ago. I have in vain tried to heal it. But the patient is in good health, although she menstruates through the fistula. I do not think that in this case or in others the fistula is attributable to the septic condition of the ligature nor indeed to the size of the ligature. Yet I am averse to large ligatures.

DR. H. BEATES, Jr.—I had one case of oophorectomy which was followed by fistula, and where healing occurred spontaneously after expulsion of a ligature.

DR. HENRY LEAMAN.—Specimen No. 1. Amelia Gerlach, aged 25 years and 3 months, unmarried, a healthy girl previous to her fifteenth year. In her sixteenth year she suffered with an inflammation of the bowels, for which she was treated by three regular physicians in consultation. She was confined to her bed for three months, and then sent to the country to gain strength. She continued to suffer from that time at every recurrence of her monthly flow, so that she has been almost constantly under medical treatment during the past nine years. Her pain at times was so severe as to bring on nervous symptoms which caused her to lose all control of herself, and she would have to be carried to bed. During the past year she was compelled to give up her occupation of housework entirely.

I saw her first about September, 1888, when she was complaining with her menstrual flow. About February 12, 1889, she came under my care with severe pains in the abdomen, which confined her to bed. These pains were paroxysmal, accompanied with chills and rise of temperature. Temperature not exceeding 103°. Urine albuminous. Counter-irritation over the lower part of the abdomen was made with a blister and poultices. While confined to bed in February, a superficial abscess formed in the left labia; after that, some swelling had occurred in left inguinal region. It was necessary to control the pain by opium suppositories and morphia powders. Examination revealed enlarged and inflamed tubes—uterus slightly movable, hypertrophied with endometritis, the mass on the right side rising two inches above the pelvis.

The patient pleaded for relief, and when an operation was proposed as the only means of permanent relief, she said that death was preferable to her continual suffering ; an unfavorable prognosis was given on account of her general condition and albuminous urine.

March 20, 1889, assisted by Drs. W. M. Welch and R. Leaman, an attempt was made to relieve her condition by an abdominal incision. The pelvis was found completely filled and impenetrable, except on the right side where a small cavity was found, from which a small mass of *débris* was removed and the wound closed. She did not rally, and died twenty-five hours after the operation. Dr. R. Leaman, who administered the ether, said that he considered her death due to the ether. She took on Cheyne-Stokes respiration while on the table. The quantity of ether taken was a half-pound can.

The *post mortem* examination showed a general inflamed condition of the ileum but no peritonitis. The mass in the pelvis could not be loosened by the hand. It was loosened by the scalpel from the brim of the pelvis. The bladder, uterus, and rectum were adherent in one mass. In the effort to remove the contents of the pelvis, a large abscess was broken on the left side of the uterus, discharging several tablespoonfuls of laudable pus. This abscess, I think, had some connection with an abscess that had appeared in the left labia previous to the operation. The end of the appendix cæci was adherent to the pelvic mass. The right kidney was contracted and hard. The spleen was atrophied entirely. The liver and kidneys were free from abscesses. The lung was not examined.

Specimens of the liver and kidneys are presented with the pelvic structures.

Specimen No. 2. Mrs. Rachel Hippansteel, aged 22; mother of one child, born October 5, 1885. Had a severe labor lasting four days. Some inflammation followed, and in two weeks she was about again. Ever since, however, she has suffered. She worked in a factory, and would have to lose several days at a time now and again. The last nine months was unable to do any work. She had frequent hemorrhages. When she would walk far, she would be seized with a severe pain in the side. On the 23d of January, 1889, she was taken with severe pain in her stomach and right leg, so that she was confined to bed; she was unable to move the right leg or walk. She was attended two weeks by a physician who said it was working on typhoid. During these two weeks she was in pain all the time, and in her own language says the pain was so bad that she pulled the hair out of her head at times. She then changed her physician, who gave her some relief with anodynes. The paroxysms of pain continued, however, to return daily. These paroxysms consisted in violent pains shooting down the right leg anteriorly, inguinal and sacro-ischiatic region.

These symptoms, which are described as nearly as possible in the patient's own language, were found on examination to originate in a localized spot of inflammation, situated in a swelling to the right of the uterus and connected with its right tube.

On the 19th of March, assisted by Drs. Hildenbrand and Drueding, the right tube and attached sac, filled with sero-pus, was removed. Her temperature just before the operation was 101°. The highest temperature after the operation was on the second day, when it reached $100\frac{3}{5}$°. The drainage tube was removed on the fourth day. The stitches were taken away, perfectly dry, on the eighth day.

On the 11th of April she wrote me, "I feel better since the operation than I have at any time since the child was born, and have no pain anywhere."

For the exact pathology of these specimens, I should like to have them referred to the society. They appear to me to be good examples of pelvic inflammation. The one, being wholly overlooked in its incipiency was progressing slowly and surely to a fatal issue. The other was wrestling with its victim on the principle of catch-as-catch-can.

DR. M PRICE: *Rupture of the Uterus.*

Was called to Mrs. B., who was suffering from rupture of the uterus in labor. The rupture took place on the left side and extended some eight inches. Child was delivered by turning; it was a cross birth, back of neck and shoulders presenting at the cervix; a large male child; it was her seventh labor; previous labors easy. She was attended by a midwife who did not see her until she had been in the labor twelve hours; she at once recognized there was something wrong, and asked the husband to get a doctor.

He called on his own physician, but he was not in ; it was my luck to be at home.

I went at once to her assistance. I examined her and found the child presented as stated; she was bleeding considerably at this time, was restless and greatly disturbed as to her safety. I decided at once to turn and deliver her ; upon passing my hand into the uterus, I found the left leg and foot outside the uterus in the peritoneal cavity.

I thought at the time it was best to deliver at once by version, and proceeded to do so. I despatched a messenger for aid, who arrived soon after the delivery, Drs. Jos. Price and W. A. Burns.

I had decided to open the patient and either stitch up the rent or remove the uterus. She was pulseless and begging for water. We could see but little hope for her, but decided to do our best, knowing she could not live in her present condition. I opened her, removed the placenta which was in the abdomen, brought the uterus out of the wound, which was slightly contracted at its fundus, with not only a long laceration in its left wall, but considerably torn from its vaginal attachment, with no apparent contractile power left.

My brother and I decided without hesitation that the best chance for the patient would be the removal of the uterus, which was at once done. The time occupied in the operation up to this time was not over five minutes ; the application of the *nœud* stopped the hemorrhage at once. The ether and injections of whiskey at this time seemed to give some promise of saving the patient, but soon after she was put back to bed, when she no longer had the stimulus of hot water to the peritoneum and the ether, she soon sank, and in thirty minutes after being put to bed was dead.

You may ask why I delayed at all in operating as soon as the discovery of rupture was made; the first and best reason was that the people could not understand a word I said, the midwife little better, and without help and advice under such circumstances one would be rash indeed to open the abdomen of any man's wife, who could see nothing wrong, save the fact that his wife was having a baby, and she had had six before; he more than likely would have thrown me out of the window,—am sure I would under the circumstances. When the matter was properly explained to him by an interpreter, he was willing to have anything done that would save his wife. I never had a case that gave me so much anxiety and trouble in so short a time.

DR. WM. GOODELL exhibited a womb in which he had performed the Cæsarean section.

The woman became pregnant while the cervix and vagina were affected with cancer. The hemorrhages had greatly reduced her, but they were in a measure controlled by the use of the curette and the actual cautery. The Cæsarean section was made on March 13, in the amphitheatre of the hospital of the university. Fourteen deep sutures and sixteen superficial ones were needed to close the uterine wound. The mother had an abundance of milk, quite enough for the nourishment of her child, and she did so well that she was to have got out of bed on April 7. But early in the morning of that day a very large hemorrhage took place from the cancerous tissues. Nothing but the tampon controlled this, and she never rallied, dying on the following day; viz., on twenty-seventh day after the operation. He had not opened the specimen, but had brought it just as it was when removed, with the parietal peritoneum adhering to the uterine cicatrix.

DR. HOWARD A. KELLY.—I was shown a very interesting specimen by Professor Zweifel, of Leipzig, last summer, removed from a patient dying early in the puerperal period.

The uterus from Dr. Goodell's case, which I am examining, is truly remarkable. In the twenty-seven days which elapsed between delivery and the woman's death, the uterus has undergone almost complete involution, and on cross-section *its walls are of uniform thickness*, and *the line of scar-tissue is invisible.* On the peritoneal surface there is a line of lymph imbedding the knots, and this, with the adhesion of the mesentery to the anterior face of the uterus, is the only naked-eye evidence that any operation has been performed.

The accuracy of the work and the excellence of Saenger's method are here wonderfully demonstrated.

Dr. WM. GOODELL also exhibited two pus-tubes as large as sausages.

The woman, aged 27, had been married three years, and was sterile. Four months after marriage she had symptoms of gonorrhœal infection; at least so Dr. Goodell inferred from her description of them, although her husband denied having ever been infected. Since that time she has had constant backache, pains in the groin, menorrhagia, and profuse leucorrhœa. In addition, she could neither work nor walk. The adhesions to the womb, broad ligament and to the rectum were close and very firm, needing very careful handling. The abdominal cavity was flushed out and a drainage tube put in. The recovery was uninterrupted.

DR. JOSEPH HOFFMAN.—This tube is almost identical with one which I removed four weeks ago to-day. There were many adhesions to the bowel. She has not made an uninterrupted recovery on account of the typhoid condition present at the operation. The tube was probably fifty per cent. larger than that exhibited to-night.

Dr. J. PRICE.—Specimens of pus-tubes as large as the uterus are quite rare. I should very much like to see this tube opened, but I am quite sure it contains pus. Some weeks ago, in the presence of certain men who take a high ground in regard to the presence of pus in these tubes, I incised tubes before the society and showed pus. Again, Dr. Goodell's statement as to the contents of the other tube should be sufficient.

A word in regard to Dr. Leaman's case. That case was treated by a prominent surgeon for bowel irritation. She has suffered the tortures of the damned. Most of these pus cases get well, no matter how many adhesions they may have. These patients are treated for everything conceivable, and finally get into the hands of one who removes large pus-tubes, and at once they become healthy and useful women. I have two cases who had been treated for typhoid fever, peritonitis, etc., by prominent men. These patients will not accept my opinion nor that of Dr. Agnew in regard to the necessity for operation, having suffered many things at the hands of many physicians.

DR. WILLIAM GOODELL.—I had expected to show another specimen, but it was, by the mistake of the nurse, put in simple water instead of in the preserving solution, and was too putrid to be brought here. I mention the condition simply on account of its rarity. It was a case in which there were five fibroid tumors of the labia,—two in one labium, and three in the other. I removed them by dissection and enucleation. In regard to the closing of the wounds, which were bleeding at many points, I debated whether to close them at once, or to stuff them with gauze, and close them on a succeeding day. I, however, closed them at once by means of several layers of cat-gut sutures, beginning at the bottom of the wounds, and gradually working upward to their edges. A drainage tube was placed on one side, and on the other a mesh of cat-gut, which acted perfectly. The parts healed by first intention without any trouble. One tumor equalled in size the largest orange. The others varied in size from that of a walnut to that of a large marble.

J. M. BALDY,
Secretary.

*328 South Seventeenth Street.*

## Figures and Tables

**Figure f1:**